# Are EDCs Blurring Issues of Gender?

**DOI:** 10.1289/ehp.113-a670

**Published:** 2005-10

**Authors:** Ernie Hood

Although scientists have postulated a wide range of adverse human health effects of exposure to endocrine-disrupting chemicals (EDCs), the nexus of the debate is the concern that prenatal and childhood exposure to EDCs may be responsible for a variety of abnormalities in human sexuality, gender development and behaviors, reproductive capabilities, and sex ratios. Scientists today are asking hard questions about potential human effects: Do EDC exposures impair fertility in men or women? Can they cause sexual organ malformations, stunted reproductive development, or testicular or breast cancer? Do fetal exposures to EDCs alter sex phenotypes? Do they change later gender-related neurobiological characteristics and behaviors such as play activity and spatial ability? Could such exposures even be involved in the etiology of children born with ambiguous gender?

EDCs include a spectrum of substances that can be loosely classified according to their known or suspected activity in relation to sex hormone receptors and pathways. The most-studied and best known are the environmental estrogens, which mimic estradiol and bind to estrogen receptors (ERs). ER agonists include the pesticide methoxychlor, certain polychlorinated biphenyls (PCBs), bisphenol A (BPA; a high production volume chemical used to make polycarbonate plastic), pharmaceutical estrogens such as diethylstilbestrol (DES) and ethinyl estradiol, and phytoestrogens, which occur naturally in many plants, most notably in soybeans in the form of genistein and related substances. There are a few known ER antagonists, or antiestrogens. Antiandrogens, or androgen receptor (AR) antagonists, include the fungicide vinclozolin, the DDT metabolite *p,p*′-DDE, certain phthalates (a group of chemicals used to soften polyvinyl chloride plastics), and certain other PCBs. And there are other types of EDCs that affect particular endocrine targets. The various EDCs differ greatly in their potencies relative to natural hormones, and in their affinity for target receptors. Some have been shown to act via non–receptor-mediated mechanisms, for example by interfering with hormone synthesis.

In many well-documented cases of high-level fetal exposures to known EDCs such as DES, certain PCBs, and DDT, the answer to the question of whether exposure is associated with gender-related effects is clearly yes. But high-level exposures such as these are relatively rare and isolated. The debate today centers on low-dose exposures—generally defined as doses that approximate environmentally relevant levels—and the idea that low-dose intrauterine exposure to some EDCs during certain critical windows of development can have profound, permanent impacts on subsequent fetal development and adult outcomes.

Critics of this idea maintain that thus far there is no credible evidence to suggest that low-dose exposures cause any adverse human health effects. But if low-dose exposures were confirmed to be the threat that proponents of the concept insist they are, public health would clearly be at risk, regulatory agencies’ risk assessment approach would need to be revised, and certain common chemicals—including some that are massively produced and economically important—would likely disappear from the marketplace.

In a June 2000 *EHP* review article on human health problems associated with EDCs, Stephen Safe, director of the Center for Environmental and Genetic Medicine at Texas A&M University, concluded that “the role of endocrine disruptors in human disease has not been fully resolved; however, at present the evidence is not compelling.” Frederick vom Saal, a developmental biologist at the University of Missouri–Columbia, disagrees, particularly in light of the research that’s been presented in the years since that review. “The jury is *not* out on human effects,” he says. “In terms of the amount of information we have in animals and the amount of information we have in humans, clearly there is a huge difference, but that’s a lot different than saying the jury is out on whether EDCs influence humans.” One thing both scientists might agree on, though, is that right now there are still more questions than answers.

## A Delicate Process

The endocrine system, comprising the hypothalamus, pituitary, testes, ovaries, thyroid, adrenals, and pancreas, is one of the body’s key communications networks. It regulates the function of specific tissues and organs by secreting hormones that act as precise chemical messengers. Development and regulation of the reproductive system is one of the major functions of the endocrine system.

Sex determination and development begin early in gestation, with the differentiation of the embryonic gonad into either testes or ovaries. If the *Sry* gene is present on the Y chromosome, it will, when activated, trigger a complex cascade of hormonal events that ultimately results in the birth of a baby boy with all of the requisite male equipment in place and functioning properly. In the absence of the *Sry* gene, the end product of the process will be a baby girl. The female phenotype is considered to be the “default” pathway for mammalian reproductive development.

Differentiation and development of the sexual organs continues throughout gestation under the guidance of the various sex hormones (such as estrogen and testosterone) produced by the endocrine system. For males and females alike, the entire process of reproductive development is exquisitely sensitive to minute changes in levels of the sex hormones, particularly during certain critical windows of development.

In papers published in the *Journal of Animal Science* throughout 1989, vom Saal demonstrated this sensitivity in a series of mouse experiments. These studies showed that in multiple-birth species it was possible for adjacently positioned male and female fetuses to transmit tiny amounts of hormones to each other, with pronounced phenotypic consequences. “We found that a difference of about a part per billion of testosterone and about twenty parts per trillion of estradiol [endogenous estrogen] actually predict entirely different brain structures, behavioral traits, enzyme levels, and receptor levels in tissues, hormonal levels in the blood—there is nothing you look for that . . . doesn’t differ in these animals,” says vom Saal.

Such a delicately timed and precisely controlled process presents a myriad of opportunities for perturbation from exposure to EDCs. These chemicals mimic hormones, and can disrupt differentiation and development in a wide variety of ways, by duplicating, exaggerating, blocking, or altering hormonal responses. The developing fetus and early neonate may lack the protective metabolic mechanisms present in adults that help detoxify and break down chemicals, maintaining homeostasis in the system. Also, tissues are rapidly dividing and differentiating in the fetus, and such a high level of cell activity is vulnerable to disruption of normal development. With such small body mass in the fetus and child compared to an adult, exposure levels may be amplified in terms of relative dosages reaching target tissues. And sometimes, exogenous EDCs may show very low binding to plasma hormone-binding proteins and thus roam the body in an unbound state, with unknown effects.

Much of what remains to be discovered about the impacts of EDC exposures on the fetus relates to a new concept called the developmental origins of health and disease (until recently known more commonly as the fetal basis of adult disease). “People are just now recognizing that this is indeed a possibility,” says NIEHS scientist Retha Newbold, a pioneer in the study of endocrine disruption who has spent decades researching the effects of exogenous estrogens, particularly DES. “Developmental exposure to low doses of EDCs may not lead to malformation or to anything you can look at and immediately recognize as a problem,” she says. “But it still could have long-term effects, such as alterations in metabolism, alterations causing cancer later on, or alterations causing infertility.”

## Evidence of Effects

Reproductive and developmental abnormalities linked to EDC exposures have now been documented in birds, frogs, seals, polar bears, marine mollusks, and dozens of other wildlife species. For example, alligators in Lake Apopka—one of Florida’s most polluted lakes due to extensive farming activities around the lake, the presence of a sewage treatment facility, and a major 1980 spill of pesticides including DDT and DDE—have been shown to have been “feminized.” That is, zoologist Louis J. Guillette, Jr., and colleagues first reported in the August 1994 *EHP*, the males have shortened penises and low levels of testosterone, while the females have excessive levels of estrogens. Sex reversal (in which an animal of one sex matures with the reproductive organs and capabilities of the other sex) and skewed sex ratios (in which there is an unusually greater proportion of one sex than the other) have been seen in several fish populations, particularly colonies living in close proximity to pulp and paper mills and sewage treatment plants. Other reports have shown reproductive effects among wildlife resulting from exposure to EDCs excreted into the water supply by women taking birth control pills.

Many of the adverse outcomes seen in wildlife populations have been replicated in laboratory experiments, confirming the role of EDCs in their occurrence. Among the papers reporting such confirmation were a May 1997 article in *EHP*, in which Guillette, D. Andrew Crain, and colleagues replicated alterations in steroidogenesis (the production of sex hormones) in alligators. More recently, in the December 2004 issue of *EHP*, Jon Nash and colleagues showed that long-term laboratory exposure to environmental concentrations of the pharmaceutical ethinyl estradiol caused reproductive failure in zebrafish.

According to a report on EDCs published in volume 75, issue 11/12 (2003) of *Pure and Applied Chemistry* by the Scientific Committee on Problems of the Environment/International Union of Pure and Applied Chemistry (SCOPE/IUPAC), more than 200 animal species are either known or suspected to have been affected by these chemicals. “The weight of evidence for endocrine disruption in wildlife is really overwhelming,” says Joanna Burger, a professor of cell biology and neuroscience at Rutgers University who cochaired the SCOPE/IUPAC project.

The SCOPE/IUPAC report was less definitive on the extent of human effects of endocrine disruptors. “It is too early to reach firm conclusions about whether human populations are seriously at risk from potential exposures to [EDCs], and further vigilance is clearly required,” the authors wrote. “However, it is somewhat reassuring that after substantial research in the past decade, there have been no conclusive findings of low-level environmental exposures to [EDCs] causing human disease.”

The report further notes, however, that “[c]hemical interferences with steroid biosynthesis and metabolism can produce adverse health effects, even though the inducing agent would not be detected as an [EDC] using receptor-based test systems. This is an important area of study because some examples of [endocrine disruption] occurring in animals derive from exposure to inhibitors of steroidogenic enzymes such as 5α-reductase and aromatase. Some such agents are known to be active in humans and are used successfully in the treatment of a range of human hormonal conditions.” The authors suggested that evaluation of such effects will require integrated screening that incorporates *in vitro* and *in vivo* technologies.

A comprehensive report issued in 2002 by the World Health Organization’s International Programme on Chemical Safety, titled *Global Assessment of the State-of-the-Science of Endocrine Disruptors*, reached similar conclusions. The report stated that “although it is clear that certain environmental chemicals can interfere with normal hormonal processes, there is weak evidence that human health has been adversely affected by exposure to endocrine-active chemicals. However, there is sufficient evidence to conclude that adverse endocrine-mediated effects have occurred in some wildlife species.” Citing the fact that studies to date examining EDC-induced effects in humans have yielded inconsistent and inconclusive results, the group wrote that, although that explains their characterization of the evidence as weak, “[that] classification is not meant to downplay the potential effects of EDCs; rather, it highlights the need for more rigorous studies.”

The *Global Assessment* further states that the only evidence showing that humans are susceptible to EDCs is currently provided by studies of high exposure levels. There is, in fact, clear evidence that intrauterine EDC exposures can alter human reproductive tract development and physiology. The most thoroughly characterized example is DES, the synthetic estrogen prescribed to millions of pregnant women in the United States and elsewhere from the 1940s to the 1970s to prevent miscarriage. The drug is known to have caused a rare form of vaginal cancer in thousands of daughters of women who took DES, as well as a variety of adverse reproductive tract effects in both the daughters and sons of those women.

The DES situation could be seen as a worst-case scenario for prenatal EDC exposure—the deliberate delivery of a potent estrogenic chemical in high doses. Viewed another way, it has provided researchers a rare opportunity to study the effects of prenatal EDC exposure in a relatively controlled fashion, with a well-defined population and well-characterized exposure to a single potent agent.

Over the course of her research, Newbold has developed a mouse model of DES exposure that has proven extremely useful in studying the effects of DES and other environmental estrogens, particularly those outcomes that may be manifested only later in life. “With the experimental model, there are a lot of questions we can ask with DES that will tell us about the weaker environmental estrogens,” she says. “We can change the timing of exposure and the amount of exposure, and we can look at different target tissues.”

The animal model has replicated numerous abnormalities reported in DES-exposed humans, and has also predicted some human outcomes. “We have published documentation [see, for example, the October 1985 issue of *Cancer Research* and volume 5, issue 6 (1985) of *Teratogenesis, Carcinogenesis, and Mutagenesis*] that a number of the reproductive anomalies seen in DES-exposed mice, such as retained testes and abnormalities in the oviduct in females, were also later reported in DES-exposed humans,” says Newbold.

## The Phthalate Connection

But reliable correlations between animal data and human outcomes have proven elusive, particularly when it comes to showing an association between human exposures to environmental EDCs at ambient levels (that is, unrelated to spills or other acute contamination events) and adverse health effects. That may be about to change for one class of chemicals—phthalates.

Phthalates are commonly used in a wide variety of consumer products such as solvents, soft plastics, and cosmetics. The National Health and Nutrition Examination Survey showed that the majority of the U.S. population carries a measurable body burden of several phthalates. There is an extensive body of literature regarding the effects of prenatal phthalate exposure in rodents. Those effects include an association between intrauterine exposure and abnormalities in male animals in a biomarker known as anogenital distance (AGD), or the distance between the rectum and the base of the penis. AGD has been shown to be a sensitive measure of prenatal antiandrogen exposure. This pattern of genital dysmorphology has come to be known as the “phthalate syndrome.”

In the first study to look at the link between AGD and EDC exposure in humans, Shanna Swan, a professor of obstetrics and gynecology at the University of Rochester, and her colleagues collected data from 85 mother–son pairs participating in the Study for Future Families, a multicenter pregnancy cohort study. The mothers’ urine was analyzed for the presence of several phthalate metabolites, and the infant boys, aged 2–36 months, were examined for genital developmental characteristics, including AGD, which was standardized for weight to develop an anogenital index (AGI).

Although the researchers found no sign of frank genital malformations or disease, they did discover an association between elevated concentrations of four phthalate metabolites in the mothers and shorter-than-expected AGI in the infants, as reported in the August 2005 issue of *EHP*. And, importantly, shortened AGI was found in infants exposed prenatally to phthalate metabolites at concentrations comparable to those found in one-quarter of the U.S. female population. The boys with short AGI were also significantly more likely to have incomplete testicular descent (cryptorchidism). “We know that incomplete testicular descent is a risk factor for poorer semen quality, lower sperm counts, [impaired fertility], and testicular cancer,” says Swan. Although it is obviously impossible to predict adult outcomes, she says these infants may be at risk of testicular dysgenesis syndrome (TDS) in the future.

TDS is a concept put forth by Danish researcher Niels Skakkebæk and colleagues, in which four adverse male reproductive end points—impaired semen quality, cryptorchidism, hypospadias (abnormal location of the urethra), and testicular cancer—are risk factors for each other. Says Swan, “The idea is that the development of the testis is interrupted in fetal life, and that this has consequences in adult life, as well as at birth. That certainly is something we’ve seen in rodents, and this study is the first evidence we’ve seen of TDS in humans.”

Swan’s study is among the first to combine a population-based, measurable, low-level EDC exposure, observed physiologic effects, and solid biological underpinnings. Even skeptic Safe says that this is the kind of study needed to begin to answer the many questions about EDCs and human health. “This looks to be a good approach, and suggests a correlation,” he says. “Whether it’s causal of anything and whether it holds up or not, I don’t know. It needs to be repeated in different locations and with more and more integrated measurements.” Swan plans to do just that, as well as to follow up on her current pregnancy cohort by measuring gender role behaviors in both the male and female children, who are now between 2 and 5 years old.

The Phthalate Esters Panel of the American Chemistry Council, a trade organization based in Arlington, Virginia, maintains that “there is no well-established and credible evidence for adverse effects [due to phthalates] in humans at environmentally relevant doses,” says panel manager Marian Stanley. With regard to Swan’s study, Stanley says, “It correlated some effects in infant males with some lower-molecular-weight phthalates, particularly diethyl phthalate, for which effects in rodents occur only at very high doses, and which is not considered to pose reproductive or developmental concerns by reviewing government agencies.”

Stanley also points to questions about the biomarker used in the study. “The measurement that was used is something that I think is still subject to debate. You see the AG distance in rodents, and while it is a marker of something, it is certainly not a biological effect,” she says. “I think the study has been overinterpreted by lots of other people [besides] the authors of the study.”

## EDCs and Sex Ratios

Sex ratio—the proportion of male to female live births—is very constant on a worldwide basis, typically ranging from 102 to 108 male births for every 100 female births. In recent years, however, a number of reports have suggested that environmental and occupational exposures to EDCs may be altering the sex ratio within given human populations.

In one such study, appearing in the July 2005 edition of *Human Reproduction*, a group of Swedish researchers analyzed blood and semen samples from 149 fishermen to investigate whether exposure to the persistent organochlorine pollutants CB-153 (a PCB) and *p,p*′-DDE affected the proportion of Y-and X-chromosome-bearing sperm. They discovered that elevated exposure levels of both chemicals were positively associated with a higher proportion of Y-chromosome sperm. The researchers conclude that their findings add to evidence that exposure to persistent organic pollutants may alter the offspring sex ratio, with the higher proportion of Y-chromosome sperm likely tending to lead to a higher proportion of male births.

A study appearing in the October 2005 issue of *EHP* takes an epidemiologic approach to the issue. Constanze Mackenzie, a member of the Faculty of Medicine at the University of Ottawa, and colleagues report a distinct skewing of the sex ratio within members of the Aamjiwnaang First Nation community near Sarnia, Ontario. They found a severe decline in the proportion of boys born among the Aamjiwnaang over the last five years, and a lesser though still significant decline over the past ten years. Although no causal factors were determined, the authors note that the community is located in immediate proximity to several large petrochemical, polymer, and chemical plants, and that previous studies—such as those following the 1976 industrial accident in Seveso, Italy—have shown that exposure to contaminants such as EDCs can impact sex ratios within small communities near such industrial facilities. The authors suggest that further assessment should be pursued to identify potential exposures among community members. [For more details on this study, see “Shift in Sex Ratio,” p. A686 this issue.]

## How Low Do They Go?

When is a hypothesis no longer a hypothesis, but a validated scientific concept ready to drive regulatory and policy decision making? When it comes to the so-called “low-dose hypothesis” regarding the biological activity or adverse effects of low-dose exposures to EDCs, that is the key question. The issue has been debated for years, since vom Saal’s group first published in the January 1997 issue of *EHP* their findings of enlarged prostate in male mice whose mothers had been fed low doses of BPA. Today, the controversy over whether vom Saal’s findings have been sufficiently replicated, and whether the U.S. Environmental Protection Agency (EPA) should revise its risk assessment process to reflect the potential for adverse effects of low-dose EDCs, is still going strong.

Some proponents of the low-dose hypothesis argue that the traditional toxicologic approach to risk assessment is an inappropriate method to assess EDCs. The current protocol assumes a linear dose-dependent response to chemical exposures, determines the lowest level at which there is an observed adverse effect, and then adds a safety factor to arrive at an official reference dose—the daily human intake assumed to be safe. Experimental work by vom Saal and others has postulated that EDCs exhibit a U-shaped dose–response curve, with biological activity stimulated at very low doses—often several orders of magnitude below current reference doses—as well as very high doses.

Proponents also state that the process of endocrine disruption itself is inherently different from many other toxicologic processes, affecting a variety of highly sensitive pathways (especially in the fetus) via novel mechanisms of action, many of which are as yet poorly understood. Also, they say, endocrine-signaling pathways that mediate responses to EDCs have evolved to act as powerful amplifiers, resulting in large changes in cell function occurring in response to extremely small concentrations.

One chemical that has become a lightning rod in the debate is BPA. By vom Saal’s count, there are now more than 100 published peer-reviewed studies showing significant biological effects of low doses of BPA (almost half published within the last two years) compared to 21 reporting no effect. He is convinced that widespread exposure to BPA poses a threat to human health.

Not so, claims Steve Hentges, executive director of the Polycarbonate Business Unit of the American Plastics Council: “For our purposes, what we have to know is, does BPA cause health effects in humans at any relevant dose, particularly at the levels at which people are actually exposed? When you look at all of the evidence together, and in particular look at the comprehensive studies that are designed to look for health effects, you don’t find them.”

The industry group also believes that the weight of evidence does not support the concept of a low-dose effect for BPA. “And it’s not just us saying that,” says Hentges. “Indeed, every government body worldwide that’s looked at it has reached effectively the same conclusion in terms of how they regulate BPA or consider regulating it.” He acknowledges that there has been quite a bit of new research activity in this area within the past few years, but states that “even though new research has been conducted, we believe that the weight of evidence has not shifted.”

Where does the EPA stand on these issues? The agency’s Office of Research and Development is in the midst of implementing a multiyear plan to set the EPA’s agenda and goals in the area of EDC research. The plan is part of the agency’s Endocrine Disruptors Research Program, a five- to ten-year research agenda it started in 2001 to look comprehensively at the science surrounding EDC exposures and effects. The integrated program was launched at about the same time that a congressional mandate, under the 1996 Food Quality Protection Act, directed the EPA to develop a screening and testing program for EDCs.

The EPA’s stance is that the jury is still out on both the public health impacts of EDCs and the need to incorporate low-dose methodologies into the agency’s risk assessment protocols. Elaine Francis, director of the Endocrine Disruptors Research Program, says the EPA needs to conduct a lot more research before any definitive public health statements can be made about this class of compounds. “When you look at such a diverse group of organisms that have been impacted in wildlife, and certainly laboratory rodent species,” she says, “there is enough concern that we recognize the importance of developing a body of work in humans to try to characterize any impact [EDCs] might be having on humans.”

The agency is currently funding three research grants in the area of low-dose EDC exposures, partly in response to the conclusions reached in a 2000 peer review and subsequent report on the low-dose issue held by the National Toxicology Program at the EPA’s request. In the 2001 *Report of the Endocrine Disruptors Low-Dose Peer Review*, that expert panel acknowledged that low-dose effects had been sufficiently documented at that point in time for the EPA to consider revisiting its current testing paradigm.

“The general consensus was that more work needed to be done in this area,” says Francis. “Since that time, we would still agree that there has not been enough information to indicate that the existing approaches are ones that would not be valid for endocrine disruptors. But we left the door open that we would need to do more research, and the best we could do at this point is to support and promote research in that area, and we’ve done that.”

Vom Saal is of a different opinion: “In the risk assessment process for chemicals as currently conducted, the maximum tolerated dose is used as a reference, and a span of typically not more than fiftyfold in the dose range is the maximum that anyone ever uses in the studies. Studies [from the 1 January 2005 issue of *Cancer Research* and the April 2005 *EHP* show] literally millions of fold below that dose range in adverse effects . . . from BPA, and when you have that type of unbelievable discrepancy, for the EPA to come out as it recently did and state that it has no intention of testing low doses as part of the testing process [implies] that you no longer have a scientifically based process—it is an entirely politically driven process, because they are explicitly ignoring the scientific findings that are out there.”

From her perspective, Newbold feels that although there is no question that EDCs have low-dose effects, more research needs to be done to document adverse effects in humans. “We spend an awful lot of time arguing whether there are low-dose effects or not. That just infuriates me,” she says. “There *are* low-dose effects. There have *always* been low-dose effects. The question is, are they adverse? We don’t know, and we’ve got to design studies to get answers to that question.” She adds, “In order to take this argument to a whole other level, we’re going to have to have more epidemiology studies. I know it happens with mice, but I don’t know what happens with humans.”

## Connecting the Gender Dots

It’s premature to call it a theory; at this point, it barely qualifies as a hypothesis: some observers are putting forth the proposition that prenatal EDC exposures may affect gender identity—how a person identifies him- or herself, regardless of physical characteristics. This idea presupposes two basic concepts: first, that transgenderism (in which a person experiences “gender dysphoria,” a strong feeling of having been born the wrong sex) is physiological in origin, most likely due to events during prenatal neurological development; second, that intrauterine EDC exposures can and do disrupt prenatal neurological development.

A paper in the 2 November 1995 issue of *Nature*, among other reports, lends credence to the first concept. Jiang-Ning Zhou and colleagues at the Netherlands Institute for Brain Research studied heterosexual men and women, homosexual men, and male-to-female transsexuals. They reported finding a distinctly female brain structure in genetically male transsexuals (men who had gone through hormonal treatment and irreversible sexual reassignment surgery to become women). The volume of the central subdivision of the bed nucleus of the stria terminalis (BSTc), a sexually dimorphic brain area that is essential for sexual behavior, is larger in men than in women. Anatomical study results showed that BSTc volume did not differ significantly between heterosexual and homosexual men, and that BSTc volume was 44% larger in heterosexual men than heterosexual women. In the male-to-female transsexuals, BSTc volume was only 52% that of the reference males—a volume analogous to that seen in the women. The authors write that these findings “support the hypothesis that gender identity develops as a result of an interaction between the developing brain and sex hormones.”

But a study by Wilson C.J. Chung and colleagues published in the 1 February 2002 *Journal of Neuroscience* complicates this picture. This group, also from the Netherlands Institute for Brain Research, reported that BSTc size differentiation between men and women became significant only in adulthood, implying that the phenomenon may be more effect than cause. The authors do point out, however, that the lack of marked sexual differentiation of the BSTc volume before birth and in childhood does not rule out early gonadal steroid effects on BSTc functions. They point to earlier animal experiments showing that fetal or neonatal testosterone levels in humans may first affect synaptic density, neuronal activity, or neurochemical content during early BSTc development, and that “[c]hanges in these parameters could affect the development of gender identity but not immediately result in overt changes in the volume or neuronal number of the BSTc.”

On the other side of the ledger, in the June 2002 edition of *EHP Supplements*, Bernard Weiss, a professor of environmental medicine and pediatrics at the University of Rochester, reviewed the existing literature on sexually dimorphic nonreproductive behaviors as indicators of endocrine disruption. Weiss made a strong evidence-based case that “gender-specific regional differentiation of the brain and, ultimately, its expression in behavior are guided by the gonadal hormones,” and that the process is subject to interference by drugs and environmental contaminants. He points out that sex differences in performance and behavior are not—but should be—a recognized criterion in developmental neurotoxicity testing.

So who out there is connecting these dots?

Scott Kerlin is a Ph.D. social scientist at the University of British Columbia. He devotes considerable time to monitoring the international scientific literature on DES and other EDCs as well as to researching and writing about the long-term health effects of pre-natal DES exposure on males. He is himself the son of a woman given DES in pregnancy.

Kerlin recently conducted a survey study of 500 members of the DES Sons International Network, an online resource for men who know or strongly suspect they were exposed to DES *in utero*. In a paper presented in August 2005 at the International Behavioral Development Symposium in Minot, North Dakota, he reports that more than 150 respondents identified themselves as having any of a variety of gender-related disorders. Kerlin does not claim that DES causes these gender disorders, but feels that his results indicate that such outcomes should be included in research related to the potential effects of prenatal EDC exposures.

## The Road Ahead

It’s going to be very difficult to ever conclusively answer the basic question of whether low-level EDC exposures during development are causing deleterious reproductive or gender-related outcomes in humans. Scientists agree that one of the major challenges is to address the issue of mixtures. Typically, researchers look at the impact of one chemical at a time, but environmental exposures regularly involve an unpredictable mix of chemicals, with exposures varying widely in dose and duration. It is unlikely there will ever be a comprehensive understanding of how the many EDCs in mixtures interact with each other and with human physiology.

Convincing epidemiologic evidence of adverse effects in humans is also difficult to come by, but will be necessary to translate scientific findings into concrete actions to protect public health. Swan’s study, one of the first of its kind to appear thus far, may serve as a methodological model for future investigations of low-level EDC exposures.

Do we know enough now that steps should be taken in the policy and regulatory realm? Some observers, taking a precautionary approach, think that we do. For example, there are bills under consideration in the California and New York legislatures to restrict the use of certain phthalates in toys, child care products, and cosmetics, and a California bill would ban the use of BPA in products meant for use by children aged 3 years or younger. Also, the European Parliament voted in 2005 to ban the use of three phthalate plasticizers (DEHP, di-*n-*butyl phthalate, and benzyl butyl phthalate) in toys and child care items, and to prohibit the use of three others (diisononyl phthalate, diisodecyl phthalate, and di-*n-*octyl phthalate) in toys and child care items that children can put in their mouths.

Theo Colborn, a professor of zoology at the University of Florida and author of the 1996 book *Our Stolen Future*, believes the time for action is now. “In the animals, it was at the population level that we really began to realize what was going on,” she says. “If we’re going to wait to see population effects for all of these concerns that we have in the human population, it’s going to be too late.” She points out that we’re already into the fourth generation of individuals who have been exposed *in utero* to chemicals that had never been used before the mid-1930s or early 1940s.

Swan agrees that there is sufficient knowledge at this point to call EDC exposures a serious threat to public health. “I don’t think it’s necessarily a threat to individuals,” she says, “but I think that as a population we are threatened. I’m not predicting the end of the species or anything like that, but I think the increasingly alarming trends that we’re seeing, in terms of couples that can’t conceive or couples whose babies have undescended testicles, and so on, can have an impact on the population as a whole.”

Other observers are not so sure. Harry Fisch, director of the Male Reproductive Center at Columbia University Medical Center, specializes in the diagnosis and treatment of male infertility. From his clinical perspective, other factors—including other exposures—are more important than EDCs. “The sky is not falling,” he says. “A lot of times there’s extrapolation from high-dose exposure to low-dose exposure. I think one of the biggest culprits for the abnormalities we see that’s been totally ignored is [increased] parental age. Also, we need to look at things we’re doing to ourselves before we start blaming low-level chemicals. For example, what does cigarette smoking do compared to Saran Wrap? What about the diets we eat, the high-fat intakes? Before we start blaming others, we need to look at ourselves to determine the impact of our lifestyles.”

Although plastic wrap may not be responsible for human infertility, the scientific evidence fueling growing concerns about the effects of ambient environmental exposures to EDCs cannot simply be dismissed. “Vigilance is the key word here, because there are so many chemicals out there,” says Burger. “Understanding the effects of chemicals is a three-pronged approach. It’s being sure that we have wildlife models and people who are watching wildlife populations to see quickly if something detrimental happens. It’s having really good epidemiological studies and vigilance of people in various places. And it’s backing those two up with laboratory science immediately when a problem turns up, to try to ascertain the cause quickly.”

## Figures and Tables

**Figure:**
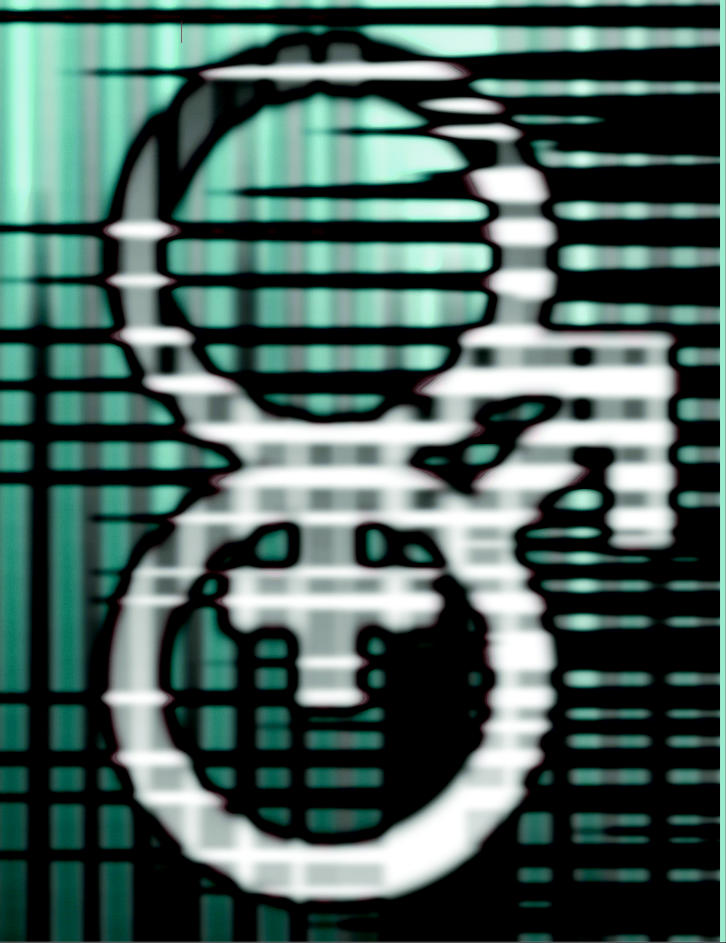


**Figure:**
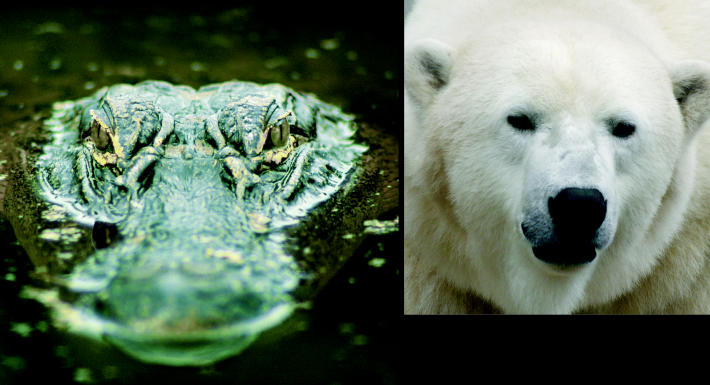
Watching wildlife. Research has documented reproductive and developmental abnormalities linked to EDC exposures in wildlife species such as alligators and polar bears, although what these results mean for humans is still unknown.

**Figure:**
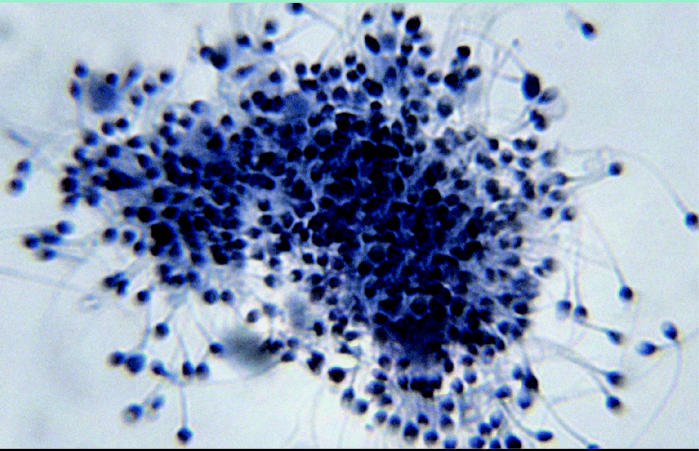
A question of Y. A Swedish study of fishermen exposed to CB-153 and *p,p*′-DDE associated elevated levels of these chemicals with a higher proportion of Y-chromosome sperm, suggesting that exposure to EDCs could skew the ratio of boys to girls.

**Figure:**
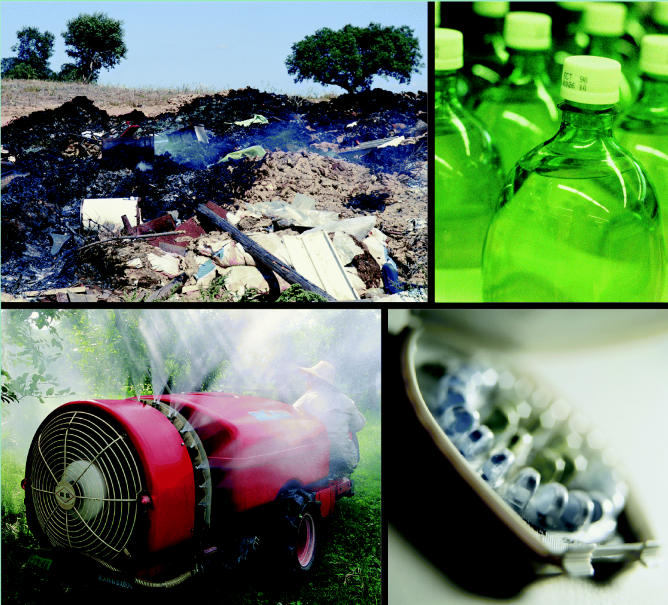
Ubiquitous exposure, unknown consequences. Humans are exposed to EDCs through many routes including pharmaceuticals, air pollution, pesticides, and drinking water, but the effects of environmental exposure are largely unknown.

**Figure:**
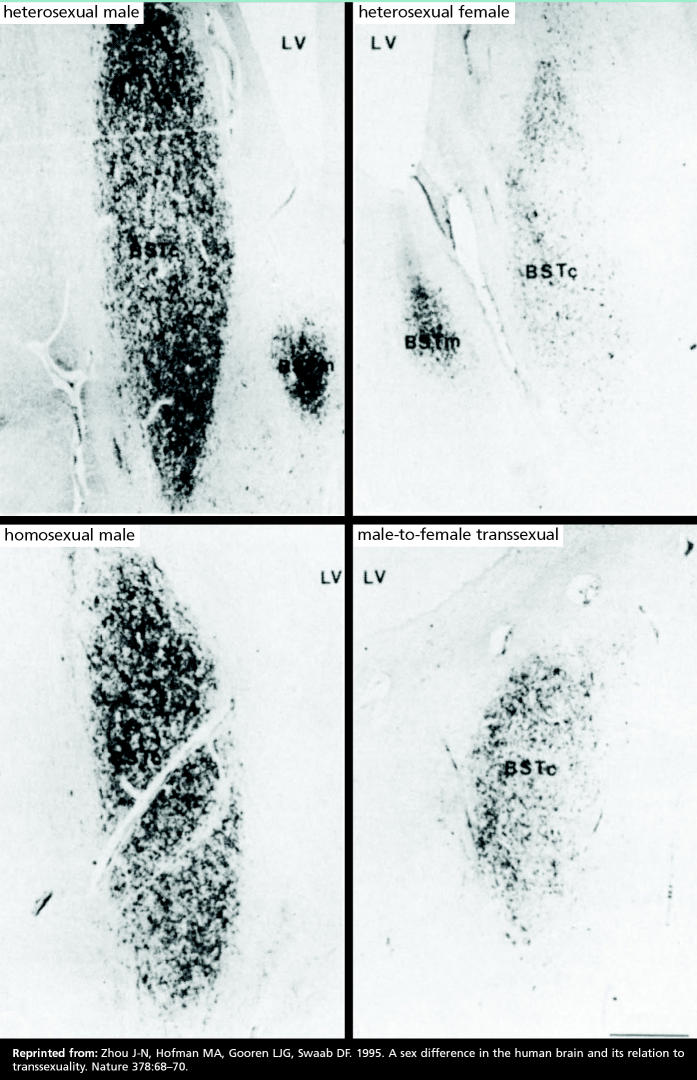
Gender basis. In a study of the brain region known as the BSTc, which varies in size by sex, the volume of the BSTc for male-to-female transsexuals was analogous to that seen in women, leading the authors to speculate that the findings “support the hypothesis that gender identity develops as a result of an interaction between the developing brain and sex hormones.”

